# Can the Posterior Segment Findings of the Eye and Serum Microbiota Metabolites Be a Biomarker in Schizophrenia?

**DOI:** 10.3390/medicina62030528

**Published:** 2026-03-12

**Authors:** Sinem Keser, Sevler Yıldız, Süleyman Aydın, Jülide Keleş, Aziz Aksoy, Elif Emre

**Affiliations:** 1 Department of Ophthalmology, Elazığ Fethi Sekin City Hospital, 23280 Elazığ, Turkey; julide_kurt@hotmail.com; 2 Department of Psychiatry, Elazığ Fethi Sekin City Hospital, 23280 Elazığ, Turkey; dr_sevler@hotmail.com; 3 Department of Biochemistry, Fırat University, 23119 Elazığ, Turkey; saydin1@hotmail.com; 4 Department of Bioengineering, Faculty of Engineering and Natural Sciences, Malatya Turgut Özal University, 44000 Malatya, Turkey; aksoy_aziz@hotmail.com; 5 Department of Anatomy, Fırat University, 23119 Elazığ, Turkey; elifkaplan1.1@gmail.com

**Keywords:** schizophrenia, TMAO, S-equol, IS, MaR1, OCT, microbiota

## Abstract

*Background and Objectives:* In many neurodegenerative diseases, the pathological changes occurring in the central nervous system may be reflected in the periphery. The aim of this study was to examine the possible relationship between the retina, choroid, and nerve fibre layer thicknesses measured on optic coherence tomography (OCT) and the serum microbiota metabolite levels of trimethyl amine-N-oxide (TMAO), S-equol, Indoxyl sulphate (IS), and Maresin 1 (MaR1). *Materials and Methods:* This study included a total of 60 subjects, comprising 30 patients diagnosed with schizophrenia and a control group of 30 healthy individuals. A sociodemographic form was given to all the subjects and the Positive and Negative Syndrome Scale (PANSS) to the schizophrenia patients. The eye fundus was evaluated with OCT. A 5 mL blood sample was taken from the arm of each subject, and the microbiota metabolite levels of TMAO, S-equol, IS, and MaR1 were examined. *Results:* The retina nerve fibre layer (RNFL) analysis results showed that the RNFL superior (*p* = 0.016), inferior (*p* = 0.002), central choroid (*p* = 0.033), nasal choroid (*p* = 0.004), temporal choroid (*p* = 0.038), and TMAO (*p* = 0.001) values were significantly lower in the schizophrenia patients than in the control group. In the patient group, a significant negative correlation was determined between the RNFL temporal measurements and IS, as well as a significant positive correlation between the central choroid measurement and the nasal choroid and temporal choroid measurements and between the nasal choroid and temporal choroid measurements. A statistically significant positive correlation was seen between S-equol and TMAO. A significant negative correlation was seen between the MaR1 level and age and disease duration. *Conclusions:* The study results showed that fundus changes are associated with serum microbiota metabolite levels in schizophrenia patients. Therefore, these parameters may be considered potential exploratory biomarkers; however, their clinical applicability requires validation in larger longitudinal studies.

## 1. Introduction

Schizophrenia is a generally destructive mental health disease with a chronic course, which has positive, negative, affective, and cognitive symptoms [1]. Although the etiopathogenesis of schizophrenia has not been fully clarified yet, attempts have been made to explain it with a neurodegeneration model [2]. With an incidence of 0.7–1% in the total population, schizophrenia is one of the most common diseases [3]. Similarly to other psychiatric diseases, schizophrenia is generally diagnosed from detailed psychiatric evaluations, and recently, neurobiological criteria that could have a positive effect on the diagnosis and treatment process have been investigated. It has even been stated that the data obtained could be used as biomarkers for schizophrenia [4].

As it is known that the retina shares embryological, anatomic, and physiological characteristics with the brain, thereby providing information about diseases that affect the brain, neurodegenerative and neuropathological changes can be examined non-invasively with optic coherence tomography (OCT). By obtaining high-resolution cross-sections, OCT is an imaging method that can evaluate tissue layers. It measures the latency and density of infrared light reflected from different tissue layers. Therefore, the choroid thickness, macula volume and thickness, and retina layer segmentation in terms of retina layers, the ganglion cell layer, the inner plexiform layer, and the retina nerve fibre layer (RNFL) can be evaluated [5,6,7,8].

It has been shown that the innermost retina layer around the optic nerve head, known as the peripapillar retina nerve fibre layer (pRNFL), is thinner in schizophrenia patients [8,9]. A 2024 meta-analysis reported that the pRNFL in schizophrenia spectrum disorders showed thinning in the macula ganglion cell layer–inner plexiform cell layer and in the retina thickness in all the other segments of the macula [10]. Research has stated that there are correlations between retina thickness in both the macula and peripapillar regions and disease duration and positive and negative symptoms [11,12]. It has also been reported that after discounting diabetes and hypertension, the retina thickness of schizophrenia patients is similar to that of healthy control subjects [13]. Although schizophrenia cannot yet be diagnosed from structural changes in the retina, it is believed that with technological advances, the clarification of this subject will be achieved with multidimensional imaging methods. In addition to conducting neuroimaging to be able to explain schizophrenia, other neurobiological parameters have been examined. Together with the metabolites produced in the intestines, when there is a decrease or change in the variety of the microbiota concept, which is used to define microorganisms living in the human organism and helps to perform physiological and biochemical functions, dysbiosis occurs [14,15]. Dysbiosis has been found to be linked with many diseases such as cancer, asthma, inflammatory bowel disease, lupus, Parkinson’s disease, multiple sclerosis, type 2 diabetes, obesity, and coronary vascular disease [16,17]. Consequently, it has been increasingly accepted that there is two-way communication between the brain and intestinal microbiota [18].

Studies have shown that intestinal microbiota have the power to produce and stimulate neurotransmitters, which are necessary for the regulation of the mental state, cognition, and general mental health [19]. One piece of evidence showing microbiota changes in schizophrenia is that there is structural damage in the gastrointestinal system and an increased intestinal response to infectious pathogens and food antigens [20]. Shi et al. reported that intestinal microbiota and metabolites could have an effect on schizophrenia pathology [21]. Various metabolites produced by intestinal bacteria may cause an inflammatory response to emerge in addition to energy metabolism [22]. The Trimethylamine N-oxide (TMAO) metabolite produced by intestinal microbiota has been shown to be associated with cardiovascular diseases [23,24]. The exposed TMAO can lead to atherosclerosis by increasing the formation of macrophage foam cells, endothelial dysfunction, thrombocyte aggregation, and vascular inflammation [25]. For S-equol, which is produced by bacterial biotypes, there is important evidence that various events can be affected not only in endothelial cells but also in vascular smooth muscle cells [26]. IS has harmful effects on the vascular system and affects many cellular functions in both endothelial cells and vascular smooth muscle cells [27]. Matsumoto et al. have explained that the role of S-equol, IS, and TMAO in vascular function stems from changes in the regulation of vascular tone, including the production of endogenous vasoactive substances, as well as alterations in the signaling pathways triggered by gut-derived substances [28]. MaR1, which is another microbiota metabolite, is produced by macrophages [29]. In a rat model, this metabolite has been shown to prevent oxidative stress and inflammation and promote apoptosis [30]. Abnormalities in the blood flow in schizophrenia can lead to neuronal–glial dysfunction, and it has been suggested that this can eventually lead to psychopathology, and vascular dysfunction has been shown to be prominent in schizophrenia [31]. In a study of fecal samples obtained from healthy control subjects and patients diagnosed with schizophrenia with the aim of showing how the intestinal microbiota and metabolites were changed in schizophrenia, the gastrointestinal and neurological results were irregular in terms of amino acid and sphingolipid metabolism in the patients [32].

The aim of the current study was to examine the links between the levels of some microbiota metabolites (TMAO, S-equol, IS, MaR1) and retina, choroid, and nerve fibre layer thicknesses measured with OCT in schizophrenia patients. Our first hypothesis is that microbiota-derived metabolites may contribute to retinal neurodegeneration via systemic inflammation and oxidative stress. Another hypothesis is that retinal morphology and microbiota metabolites may be linked through a common systemic inflammatory and vascular pathway. Analyses were also performed to determine whether there was a relationship between both the OCT measurements and the microbiota metabolite levels found using quantitative methods in the schizophrenia patients. It was considered that the determination of this relationship would shed light on the clarification of the neurodegenerative process in the etiopathogenesis of schizophrenia and the determination of the connection with inflammation formed in the disease process.

## 2. Material and Method

The patient group was formed of 35 patients who presented at the Psychiatry Clinic of Fethi Sekin City Hospital between October 2024 and March 2025; were diagnosed with schizophrenia; met the study criteria; and, following evaluation by a psychiatry specialist, were evaluated by a specialist in ophthalmology diseases. Sample size calculation was performed using G*Power software (version 3.1.9.2, Heinrich-Heine-Universität Düsseldorf, Düsseldorf, Germany), based on the study titled “Retinal Nerve Fiber Layer Structure Abnormalities in Schizophrenia and Its Relationship to Disease State: Evidence From Optical Coherence Tomography.” According to the power analysis, a minimum total sample size of 60 participants, comprising at least 30 patients and 30 healthy controls, was required to achieve a 95% confidence level and 95% statistical power. The group of patients diagnosed with schizophrenia consisted of those who had not used any psychiatric medication in the past 6 months and had discontinued treatment with antipsychotics or other psychotropic drugs. The 6-month period was considered sufficient to assess the potential effects of drug treatment on the retina and microbiota. The healthy control group was formed of 32 healthy individuals who presented at the same hospital for a routine yearly check-up and had no medical problem. Five patients diagnosed with schizophrenia and two control group subjects later withdrew from this study on their own request, leaving 30 subjects in each group for evaluation.

Structured interviews according to the DSM-5, each lasting approximately 30 min, were conducted by a psychiatrist. The study exclusion criteria were defined as not responding to the questions according to the diagnostic criteria questioned in this study, illiteracy, being outside the age range of 18–65 years, a history of systemic disease including type 2 diabetes and hypertension, the presence of any eye disease or a history of eye surgery for any reason. Written informed consent was provided by all the study participants or their guardian, if appropriate.

The sociodemographic data form was completed by all the participants and the Positive and Negative Syndrome Scale (PANSS) by the schizophrenia patients; then biological samples were obtained from the patient and control groups. A full ophthalmological examination [visual acuity, intraocular pressure, biomicroscopic and detailed fundus examination] was performed together with OCT examination in the Ophthalmology Clinic.

### 2.1. Scales Used in This Study

Sociodemographic and Clinical Data Form: This form was prepared by the researchers, taking into consideration the aims of this study and according to the information obtained from the scanned resources and clinical experience.

Positive and Negative Syndrome Scale (PANSS): This scale was developed by Kay et al. to evaluate positive and negative symptoms and general psychopathology in patients with schizophrenia or other psychotic disorders [33]. Validity and reliability studies of the Turkish version of the scale have been conducted [34].

**Figure 1:**
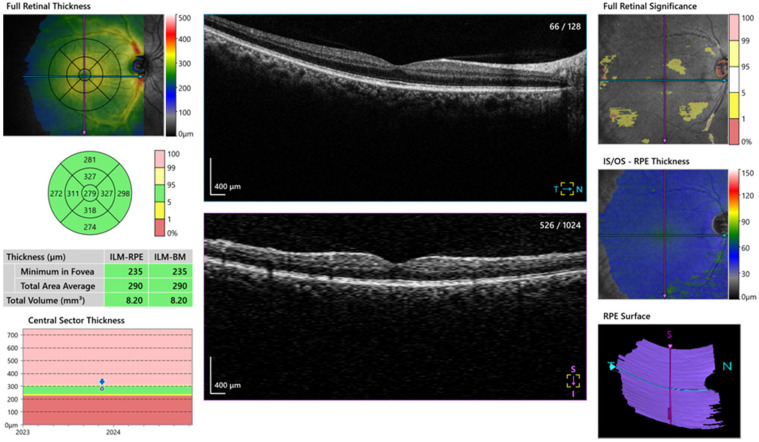
Central macula thickness (CMT).

**Figure 2:**
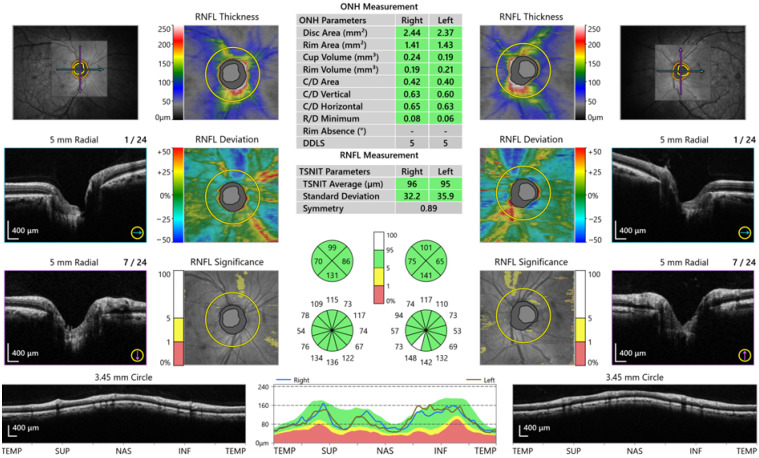
Retina nerve fibre layer (RNFL).

All the study participants underwent a full ophthalmological examination. Images of the macula, RNFL and choroid layer were obtained with a Spectral-Domain AS-OCT device (OCT-HS100, Canon Inc., Tokyo, Japan). This OCT device has 3 mm axial resolution and 2 mm scanning depth at a 70,000 A-scan/sec scanning speed. During the measurements, the choroid mode providing a high-quality image was used. The central macula thickness (CMT) (Figure 1) and the RNFL (Figure 2) were automatically analyzed in 4 quadrants on the OCT device.

**Figure 3:**
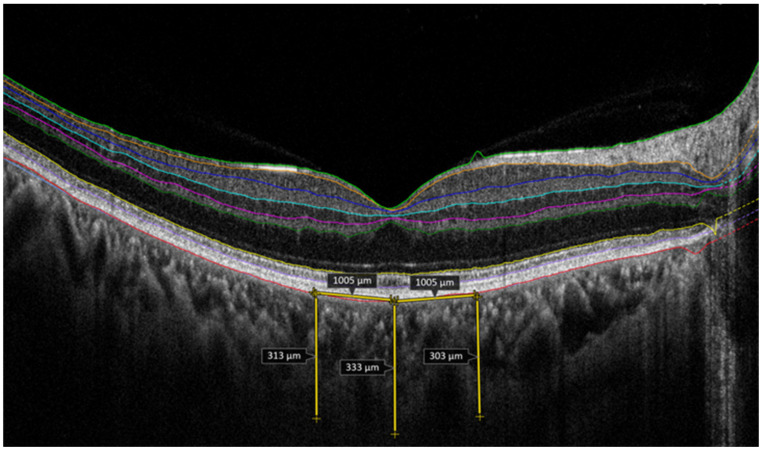
Central, temporal and nasal choroid thickness.

Choroid thickness was measured vertically between the inner surface of the sclera and the outer border of the hyper-reflective line corresponding to the RPE (Figure 3). The temporal and nasal choroid thicknesses were measured at a distance of 1000 µm from the fovea (Figure 3). For all cases, only the right eye OCT measurements were included in the statistical analyses to ensure methodological consistency. All the OCT images were evaluated separately by two researchers, and a mutual decision was reached through comparisons and discussion.

### 2.2. Analysis of Biological Samples

Fasting blood samples were taken from the antecubital vein of patients and healthy controls in the sitting position. Then, they were centrifuged at 4000 rpm for 5 min. They were stored at −20 degrees until used. Serum TMAO (Human TMAO, catalogue no: 201-12-7378 Sunred Biotech Co., Ltd., Shangai, China), S-equol apelin (Human S-equol, catalogue no: 201-12-8142 Sunred Biotech Co., Ltd., Shangai, China), IS apelin (Human IS, catalogue no: 201-12-7596 Sunred Biotech Co., Ltd., Shangai, China), and MaR1 apelin (Human MaR1, catalogue no: 201-12-7339 Sunred Biotech Co., Ltd., Shangai, China) were studied by the ELISA method according to the manufacturer’s instructions provided in each kit. The measurement ranges of the TMAO, S-equol, IS, and MaR1 biomarkers were 0.05–10 ng/mL, 0.25–70 ng/mL, 2–600 µg/mL, and 7.5–2000 pg/mL; sensitivities were 0.043, 0.247, 1.854, and 7.247, respectively. The intra- and interassay CV values of the TMAO, S-equol, IS, and MaR1 biomarkers were <10–<12%, <10–<12%, <10–<12% and <10–<12%, respectively. A Bio-Tek ELX50 [BioTek Instruments, Winooski, VT, USA; Human Diagnostics] automatic plate washer was used for plate washing, and ChroMate and Microplate Reader P4300 devices (Awareness Technology Instruments, Palm City, FL, USA) were used for absorbance readings.

### 2.3. Statistical Analysis

The data obtained in this study were analyzed statistically using SPSS vn. 22 software (Statistical Package for Social Sciences; SPSS Inc., Chicago, IL, USA). The conformity of continuous data to a normal distribution was examined with the Kolmogorov–Smirnov test. Descriptive statistics were stated as the mean ± standard deviation (SD) or median and interquartile range (IQR: 25th–75th percentile) values for continuous variables and as the number (n) and percentage (%) for categorical variables. In the comparisons of categorical variables between groups, Pearson Chi-square analysis was used. In the comparisons of paired groups of continuous variables showing normal distribution, Student’s *t*-test was applied, and when distribution was not normal, the Mann–Whitney U-test was used. In the examination of relationships between continuous variables, Pearson correlation analysis was applied to data showing normal distribution and Spearman correlation analysis when distribution was not normal. A Receiver Operating Characteristic (ROC) curve was drawn to measure the diagnostic value of parameters for schizophrenia. A multivariate logistic regression analysis was performed to evaluate the independent predictors of schizophrenia after adjusting for potential confounding variables, including age, sex, and smoking status. Statistical significance was defined as a two-tailed *p* value < 0.05 in all analyses.

## 3. Results

Evaluations were made of a total of 60 study participants, comprising 30 schizophrenia patients and 30 healthy control subjects. Females constituted 6.7% of the patient group and 10% of the control group with no significant difference between the groups (*p* = 0.999). The mean age was 44.8 ± 12.3 years in the patient group and 44.5 ± 12.7 years in the control group, with no significant difference between the groups (*p* = 0.934). All the patients selected were not using psychiatric drugs. There was a history of attempted suicide in 20% of the patient group, and disease duration was calculated to be mean 20.4 ± 10.1 years (Table 1).

The RNFL superior (*p* = 0.016), RNFL inferior (*p* = 0.002), central choroid (*p* = 0.033), nasal choroid (*p* = 0.004), temporal choroid (*p* = 0.038), and TMAO (*p* = 0.001) values were determined to be significantly lower in the schizophrenia patient group than in the control group (Table 2).

A statistically significant negative correlation was seen between the RNFL temporal measurement and IS. A statistically significant positive correlation was determined between the central choroid measurements and the nasal choroid and temporal choroid measurements, between the nasal choroid measurement and the temporal choroid measurement, and between the S-equol and TMAO levels. The MaR1 level was seen to be significantly negatively correlated with age and disease duration (Table 3).

**Figure 4:**
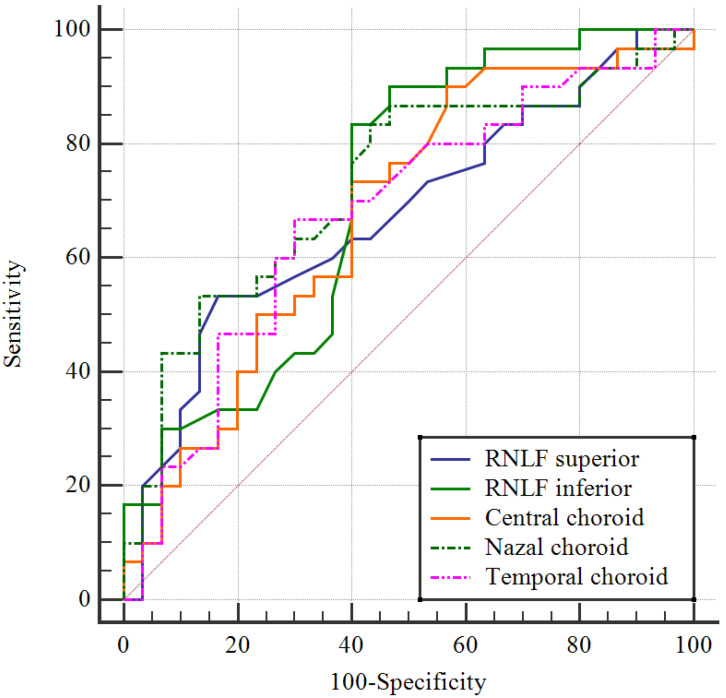
The ROC curve of the OCT images for schizophrenia disease.

**Figure 5:**
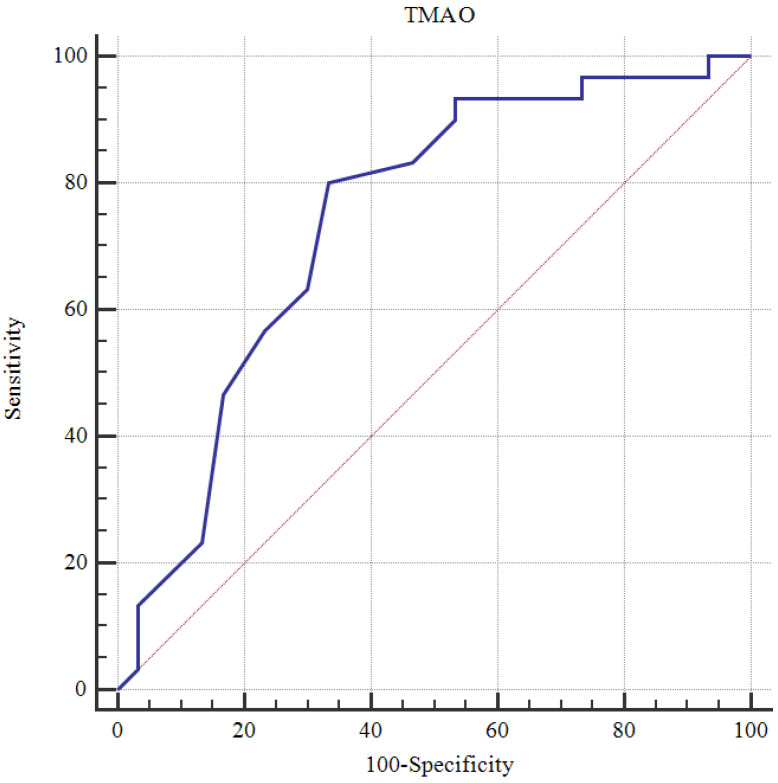
The ROC curve of the TMAO values for schizophrenia disease.

ROC analysis was performed, and cutoff values were determined for the ability of values to predict schizophrenia. The cutoff values demonstrating good markers were determined to be 117 for the RNFL superior with 53.3% sensitivity and 83.3% specificity, 133 for the RNFL inferior with 83.3% sensitivity and 60% specificity, 416 for the central choroid with 90% sensitivity and 43.3% specificity, 305 for the nasal choroid with 53.3% sensitivity and 86.7% specificity, 357 for the temporal choroid with 66.7% sensitivity and 70% specificity (Table 4, Figure 4), and 1.2 for TMAO with 80% sensitivity and 66.7% specificity (Table 4, Figure 5).

Because the prevalence of smoking differed significantly between the groups, a multivariate logistic regression analysis was performed to control for the potential confounding effect. Age, sex, and smoking status were included in the model as covariates, and retinal parameters and serum metabolites found to be significant in the univariate analyses were added to the model. The model was found to be statistically significant overall (Omnibus test, *p* < 0.001) and showed good fit (Hosmer–Lemeshow *p* = 0.992); according to the Nagelkerke R^2^ value, it explained 87.9% of the variance. After adjustment, central choroidal thickness (OR = 0.957; 95% CI: 0.921–0.994; *p* = 0.024) remained independently associated with schizophrenia, whereas inferior RNFL thickness and TMAO did not maintain statistical significance (Table 5).

## 4. Discussion

To be able to better understand the neurobiology of schizophrenia patients, the retina and related areas were evaluated with OCT, and serum TMAO, S-equol, IS, and MaR1 levels were measured to evaluate some metabolites in the intestinal microbiota, and all these parameters were compared with those of a healthy control group. The results of this study showed that in the schizophrenia patient group, in addition to thinning in some regions of the retina structure, there was thinning in the choroid, which has an important role in providing oxygen and nutrients to the outer retina, which is one of the most vascularized tissues in the human body. In the serum microbiota metabolites, the TMAO values were seen to be significantly lower in the patient group, which indicated that this should be evaluated as a biomarker in schizophrenia.

Advances in technology are increasingly contributing to the clarification of morphological changes in the retina in patients with schizophrenia. Pan et al. reported that the mean RNFL thickness was significantly reduced in schizophrenia patients compared to healthy control subjects [35]. A decrease has been determined in the volume of structural grey matter in the brain on magnetic resonance imaging of schizophrenia patients experiencing a first attack, and there has been shown to be a decrease in RNFL and macula thickness compared to control subjects [36]. In a recent systematic examination and meta-analysis of schizophrenia patients, there was shown to be a decrease in the mean macula thickness, macula ganglion cell–inner plexiform layer thickness, and macula volume [10].

However, in contrast to these findings, in a study that included 38 schizophrenia patients, Chu et al. reported that all the retina RNFL thickness and macula volume values were similar in patients and control subjects (*p* = 0.86 and *p* = 0.64, respectively). It was suggested that as the positive symptom severity was associated with a smaller macula volume, consequently, the myelinolysis of axons was not affected in schizophrenia patients because of the normal macula volume and RNFL thickness [37]. Silverstein et al. also found no difference in the RNFL, macula or ganglion cell–inner plexiform layer thickness in schizophrenia patients and attributed the thinning in these layers to the presence of diabetes or hypertension in the sample in general [13]. In the current study, patients with comorbidities such as hypertension or diabetes were not included, and the RNFL superior (*p* = 0.016), RNFL inferior (*p* = 0.002), central choroid (*p* = 0.033), nasal choroid (*p* = 0.004), and temporal choroid (*p* = 0.038) values were determined to be significantly lower than those of the control group. This change in the retina can be evaluated as a sign of axonal degeneration, which may be responsible for a decrease in grey matter volume and thinning in areas related to the retina.

It has recently been discussed that the interaction between intestinal microbiota and microbial metabolites may affect brain function through the intestine–brain axis, and thus there may be a predisposition to the development of schizophrenia [21]. Liang et al. reported that a predisposition to schizophrenia was created by glycerophospholipids and fatty acid metabolism mainly affecting intestinal microbiota [38]. In pre-clinical pioneering studies, Li et al. determined some metabolites in schizophrenia patients [39]. In another study of 41 acute schizophrenia patients and 39 schizophrenia patients in remission, a 16S ribosomal RNA (16SrRNA) gene sequence-based approach and a non-targeted liquid chromatography–mass spectrometry-based approach were used to measure the intestinal microbiome and microbial metabolites, and evaluations were made regarding whether or not microbial dysbiosis and microbial metabolite biomarkers were associated with the severity of schizophrenia symptoms. Compared to the healthy control subjects, a change in the intestinal microbial composition of the patients suggested that intestinal microbiota and metabolites could have a potential role in the pathophysiology of schizophrenia.

As there are few studies in the literature that have examined the relationship between schizophrenia and microbiota, the serum levels of intestinal microbiota metabolites were measured in the current study. In this respect, this is the first such study in the literature. The results showed that the serum TMAO values of the schizophrenia patients were significantly lower than those of the control group. Previous studies have reported elevated plasma TMAO levels in patients with type 2 diabetes, atherosclerotic plaque accumulation, and peripheral artery disease [40,41]. Recent studies have also shown that increased plasma TMAO levels in circulation are closely related to metabolic disorders and contribute to endothelial dysfunction [42]. The risk of cardiovascular disease is known to be increased in schizophrenia patients [43], and this difference from the literature may be due to the small sample size in the current study. However, a significant negative correlation was determined between the RNFL temporal measurement and the serum IS level in the patient group. Elevated serum concentrations of IS in dialysis patients have been previously found to be associated with cardiovascular damage and low survival rates [44]. A decrease in retina thickness in schizophrenia suggests that there may be vascular changes. The current study results showed a significant negative correlation between the MaR1 level and age and disease duration. Shaalan et al. [45] similarly reported that this negative correlation of MaR1 in long-term schizophrenia suggested that it could be a negative regulator of the inflammatory process.

In our study, age, gender, and smoking status were included in the statistical model to account for potential confounding effects. Smoking was shown to have an effect on vascular parameters, retinal thickness, and circulating metabolites [46,47]; therefore, these factors are important for understanding the pathophysiology of schizophrenia and related retinal changes. Including smoking status in the model helped control for the potential effects of smoking on the results. After this analysis, central choroidal thickness continued to be significantly associated with schizophrenia (OR = 0.957; 95% CI: 0.921–0.994; *p* = 0.024), whereas lower RNFL thickness and TMAO levels did not show statistical significance. Our findings suggest that central choroidal thickness may be a more reliable biomarker for schizophrenia diagnosis when smoking status is taken into account. However, it should not be overlooked that smoking itself may contribute to vascular and metabolic changes that are not fully captured in this model. Longitudinal studies evaluating smoking duration and intensity are needed; this would provide a clearer explanation of how smoking may affect retinal and metabolic changes in schizophrenia.

Strong aspects of this study were that both the OCT findings and microbiota metabolite levels were evaluated together and potential relationships were examined to be able to understand the pathogenesis of schizophrenia disease, in which there remain many neurobiological uncertainties. We based our microbiome assessment solely on serum metabolite measurements, which provided indirect information about microbiome activity. This approach was preferred due to its feasibility and non-invasive nature, and it allows for data collection in large samples. While serum metabolite profiling can provide valuable information about systemic metabolic changes, it does not directly capture the diversity or composition of the gut microbiome. Future studies could integrate direct microbiome sequencing techniques (e.g., 16S rRNA sequencing) to enable a more detailed and accurate characterization of the microbiota. These analyses may enable a comprehensive analysis of microbial diversity, functional capacity, and specific microbiota-related pathways contributing to retinal neurodegeneration, as well as the identification of more accurate biomarkers in conditions such as schizophrenia.

However, there were also some limitations to this study, primarily the small sample size; the cross-sectional, single-centre design; the fact that the majority of the patients were active smokers; and the fact that the intestinal microbiota were evaluated by examining the serum microbiota metabolite level values. The cross-sectional design of this study makes it difficult to fully assess the causal effects of microbiota-derived metabolites on retinal neurodegeneration. We hypothesized that microbiota-derived metabolites may contribute to retinal neurodegeneration via systemic inflammation and oxidative stress and that retinal morphology and microbiota metabolites may be linked through a common systemic inflammatory and vascular pathway. Since both hypotheses suggest that the relationship between retinal changes and the microbiota is multifactorial and develops over time, long-term studies are important. This study included patients diagnosed with schizophrenia who had not used any psychiatric medication in the past 6 months. However, the long-term use of psychotropic medications can cause permanent changes in retinal morphology and microbiota metabolite levels. Therefore, the 6-month period only allows for the observation of short-term effects, and the potential effects of longer-term medication use have been overlooked. Once again, the cutoff values obtained in the ROC analysis provide interesting findings for predicting schizophrenia, but there are some limitations regarding whether they are sufficient for clinical screening. For example, the sensitivity for the superior RNFL is low (53.3%), while the specificity is high (83.3%), which may lead to the exclusion of patients in the early stages. Similarly, the specificity of the TMAO value (66.7%) is also limited. At this stage, the use of these parameters as auxiliary research tools is more appropriate, and they do not appear to be sufficient for clinical diagnosis alone. In the future, validation studies in larger populations and different disease groups are needed.

## 5. Conclusions and Recommendation

As a result of this study, in which multidisciplinary evaluations were made regarding peripheral findings of schizophrenia, it was concluded that there is a need for further longitudinal studies to monitor the changes in the retinal layers throughout the course of the disease and to examine a greater number of parameters to be able to define the intestinal microbiota, which is a broad flora. Our findings regarding retinal changes and abnormalities in microbiota metabolite levels in schizophrenia patients are related to the progressive nature of this disease and indirectly provide clues about neuroprogression and neurodegeneration processes. This situation emphasizes the importance of monitoring neurological functions. Investigating how schizophrenia affects neuroprogression or neurodegeneration processes and its relationship with these indicators will contribute to the personalization of treatment strategies. Patients who had not used any psychiatric medication in the past 6 months were included. However, previously used psychotropic medications may affect retinal morphology and microbiota metabolite levels. Long-term antipsychotic treatment may cause changes in retinal structure and leave lasting effects on the microbiota. Therefore, the effect of past medication use on these parameters should be examined in more detail. The clarification of these parameters is important in terms of their use as biomarkers, which would enable the early diagnosis of schizophrenia before the emergence of disease symptoms, as early diagnosis and treatment are known to reduce morbidity and mortality rates, and in terms of evaluating well-being during the course of the disease. Thus, this study can be considered of value not only for psychiatrists to understand the pathogenesis of schizophrenia, which is a neurodegenerative disease, but also for the early detection of schizophrenia disease by other clinical branches.

## Figures and Tables

**Table 1:** Comparison of demographic and clinical characteristics between groups.

		Patients Group (n = 30)		Controls Group (n = 30)		*p*
		n	%	n	%	
Gender	Female	2	6.7	3	10.0	0.999 *
	Male	28	93.3	27	90.0	
Age (years), mean ± SD		44.8 ± 12.3		44.5 ± 12.7		0.934 **
Marital status	Single	21	70.0	14	46.7	0.067 *
	Married	9	30.0	16	53.3	
Education level	Primary school	17	56.7	12	40.0	0.174 *
	High school	9	30.0	8	26.7	
	University	4	13.3	10	33.3	
Place of residence	Rural	5	16.7	6	20.0	0.739 *
	Urban	25	83.3	24	80.0	
Employment status	Employed	4	13.3	7	23.3	0.317 *
	Unemployed	26	86.7	23	76.7	
Smoker	Yes	23	76.7	12	40.0	0.004 *
	No	7	23.3	18	60.0	
Alcohol consumption	Yes	0	0.0	0.0	0	^-^
	No	30	100.0	30	100.0	

* Chi-square test was used for categorical variables. ** Student’s *t*-test was used for continuous variables.

**Table 2:** Comparison of retinal, choroidal, and serum metabolite parameters between groups.

	Patient Group (n = 30)	Control Group (n = 30)	*p*
	Mean ± SD	Mean ± SD	
Macula	268.4 ± 19.9	276.3 ± 21.7	0.149 *
Central macula, Mean ± SD	120.0 ± 14.3	129.6 ± 15.8	0.016 *
RNFL superior, Mean ± SD	121.6 ± 14.7	135.9 ± 19.8	0.002 *
RNFL inferior, Mean ± SD	73.2 ± 8.4	74.7 ± 11.8	0.566 *
RNFL temporal, Mean ± SD	83.0 ± 9.8	88.2 ± 12.0	0.075 *
RNFL nasal, Mean ± SD	378.0 ± 59.0	411.6 ± 60.7	0.033 *
Central choroid, Mean ± SD	315.3 ± 55.3	356.8 ± 50.6	0.004 *
Nasal choroid, Mean ± SD	350.7 ± 51.0	379.9 ± 55.4	0.038 *
Temporal choroid, Mean ± SD	9.1 (6.3–11.4)	1.1 (0.1–15.4)	0.179 **
S-equol, Median (IQR)	1.0 (0.9–1.2)	1.4 (1.1–2.6)	0.001 **
TMAO, Median (IQR)	122.9 (103.1–398.7)	111.8 (95.4–231.5)	0.379 **
IS, Median (IQR)	18.0 ± 11.4	4.7 ± 4.1	<0.001 *
MaR1, Median (IQR)	0.88 (0.71–1.60)	0.77 (0.59–1.49)	0.352 **

* Student’s *t*-test was used. ** Mann–Whitney U test was used. RNFL: retina nerve fibre layer; TMAO: trimethyl amine-N-oxide; IS: indoxyl sulphate; MaR1: maresin-1.

**Table 3:** Correlation analyses in the schizophrenia patient group.

		PANNS	Central Macula	RNFL Superior	RNFL Inferior	RNFL Temporal	RNFL Nasal	Central Choroid	Nasal Choroid	Temporal Choroid	S-Equol	TMAO	IS	MaR1
Central macula	r	0.350												
	*p*	0.058												
RNFL superior	r	−0.057	−0.111											
	*p*	0.765	0.559											
RNFL inferior	r	−0.278	−0.066	0.272										
	*p*	0.137	0.727	0.145										
RNFL temporal	r	−0.109	−0.226	−0.138	0.129									
	*p*	0.567	0.230	0.466	0.495									
RNFL nasal	r	0.016	−0.102	−0.164	0.036	−0.186								
	*p*	0.931	0.590	0.386	0.852	0.325								
Central choroid	r	−0.111	−0.218	0.036	−0.029	0.214	−0.148							
	*p*	0.558	0.248	0.850	0.881	0.255	0.434							
Nasal choroid	r	−0.042	−0.111	−0.042	0.041	0.216	−0.060	0.844						
	*p*	0.826	0.559	0.825	0.829	0.252	0.754	<0.001						
Temporal choroid	r	−0.232	−0.144	0.127	0.111	0.335	−0.173	0.771	0.702					
	*p*	0.218	0.448	0.503	0.560	0.070	0.360	<0.001	<0.001					
S-equol	r	−0.334	−0.076	0.074	0.105	−0.238	0.361	−0.127	−0.249	−0.142				
	*p*	0.071	0.690	0.697	0.581	0.205	0.050	0.505	0.185	0.454				
TMAO	r	0.020	−0.103	0.284	−0.029	−0.344	0.256	−0.158	−0.180	−0.208	0.457			
	*p*	0.915	0.588	0.128	0.878	0.063	0.172	0.403	0.340	0.269	0.011			
IS	r	−0.262	0.066	0.225	0.252	−0.466	−0.130	−0.239	−0.142	−0.243	0.115	0.258		
	*p*	0.163	0.730	0.232	0.178	0.010	0.494	0.203	0.453	0.196	0.545	0.169		
MaR1	r	−0.093	0.154	0.296	0.165	−0.084	−0.229	0.160	0.121	0.056	0.001	0.127	0.121	
	*p*	0.624	0.416	0.112	0.384	0.659	0.224	0.397	0.525	0.771	0.994	0.504	0.522	
Age	r	0.196	−0.234	−0.251	−0.066	0.217	0.169	−0.185	−0.114	−0.091	−0.198	−0.275	−0.331	−0.619
	*p*	0.300	0.214	0.182	0.730	0.249	0.373	0.327	0.547	0.632	0.294	0.142	0.074	<0.001
Disease duration	r	0.120	−0.183	−0.128	0.211	0.074	0.241	−0.054	0.043	0.101	−0.250	−0.317	−0.185	−0.461
	*p*	0.529	0.334	0.499	0.264	0.698	0.199	0.777	0.822	0.597	0.183	0.088	0.328	0.010

**Table 4:** The sensitivity and specificity values of the measured parameters in the determination of a diagnosis of schizophrenia.

	Area	*p*	95% Confidence Interval		Sensitivity	Specificity	PPV	NPV
			Lower Limit	Upper Limit				
RNFL sup ≤ 117	0.676	0.012	0.543	0.791	53.3	83.3	76.2	64.1
RNFL inf ≤ 133	0.709	0.002	0.578	0.819	83.3	60	67.6	78.3
Central choroid ≤ 416	0.678	0.011	0.545	0.793	90	43.3	61.4	81.2
Nasal choroid ≤ 305	0.729	0.001	0.599	0.836	53.3	86.7	80	65
Temporal choroid ≤ 357	0.677	0.012	0.544	0.792	66.7	70	69	67.7
TMAO ≤ 1.2	0.742	<0.001	0.613	0.847	80	66.7	70.6	76.9

PPV: positive predictive value; NPV: negative predictive value.

**Table 5:** Multivariable logistic regression analysis of factors associated with schizophrenia.

	B	S.E.	*p*	OR	95% CI for OR	
					Lower	Upper
RNFL inferior	−0.093	0.051	0.065	0.911	0.825	1.006
Central choroid	−0.044	0.020	0.024	0.957	0.921	0.994
TMAO	−1.746	0.932	0.061	0.175	0.028	1.084
Age	−0.279	0.130	0.031	0.757	0.587	0.975
Male gender	0.931	2.364	0.694	2.537	0.025	260.799
Smoker	5.106	2.209	0.021	165.026	2.173	12,533.611

The multivariable logistic regression analysis was adjusted for age, sex, and smoking status. OR: odds ratio; CI: confidence interval.

## Data Availability

The data sets used and/or analyzed during the current study are available from the corresponding author on reasonable request.
